# Medication optimization according to the Fit fOR The Aged (FORTA) rules improves functional status in patients hospitalized for geriatric rehabilitation

**DOI:** 10.1007/s41999-023-00779-w

**Published:** 2023-04-19

**Authors:** Farhad Pazan, Martin Wehling, Christel Weiss, Helmut Frohnhofen

**Affiliations:** 1 Clinical Pharmacology Mannheim, Medical Faculty Mannheim, Ruprecht-Karls-University Heidelberg, Theodor-Kutzer-Ufer 1-3, 68167 Mannheim, Germany; 2 Department of Medical Statistics, Biomathematics and Information Processing, Medical Faculty Mannheim, Heidelberg University, Mannheim, Germany; 3 Faculty of Health, Department Medicine, University Witten-Herdecke, Alfred-Herrhausen-Str. 50, 58455 Witten, Germany; 4 Department of Orthopedics and Trauma Surgery, Medical Faculty, Heinrich-Heine-University Duesseldorf, Moorenstr. 5, 40225 Duesseldorf, Germany

**Keywords:** FORTA score, Activities of daily living, Randomized clinical trial, Polypharmacy, Older people

## Abstract

**Introduction:**

Functional status is one of the most important issues of geriatric care. Polypharmacy seems to be a modifiable factor associated with functional decline in older adults. However, the impact of pharmacotherapy optimization on the activities of daily living in patients undergoing geriatric rehabilitation has not been investigated prospectively so far.

**Methods:**

This post hoc analysis of a subsample of the VALFORTA study included individuals only undergoing geriatric rehabilitation with a length of in-hospital stay of at least 14 days. Medication was modified according to the FORTA rules in the intervention group while in the control group standard drug treatment was applied. Both groups received comprehensive geriatric treatment.

**Results:**

The intervention and control groups consisted of 96 and 93 individuals respectively. They did not differ according to basic data except for age and Charlson Comorbidity Index (CCI) on admission. On discharge, activities of daily living (Barthel index, BI) were improved in both groups. An increase of at least 20 points of the BI was observed in 40% of patients in the intervention group and in 12% of patients in the control group (*p*< 0.001). Logistic regression analysis with an increase of at least 20 BI-points was significantly and independently associated with patient group (2.358, *p*< 0.02), BI on admission (0.957, *p*< 0.001), and the CCI (0.793, *p*< 0.041).

**Conclusion:**

This post hoc analysis of a subsample of older individuals hospitalized for geriatric rehabilitation demonstrates a significant additional improvement in activities of daily living by modification of medication according to FORTA.

**Registration:**

DRKS-ID: DRKS00000531.

## Key summary points

**Aim:**

To analyze the impact of medication optimization according to the FORTA list on functional status in patients undergoing geriatric rehabilitation.

**Findings:**

An increase of at least 20 points of the BI was observed in 40% of patients in the intervention group and in 12% of patients in the control group (*p*< 0.001). Logistic regression analysis with an increase of at least 20 BI-points was significantly and independently associated with patient group (2.358, *p*< 0.02).

**Message:**

Medication optimization according to the FORTA rules has a significant additional improvement in activities of daily living in older adults hospitalized for geriatric rehabilitation.

## Introduction

Functional status is one of the most important determinants of geriatric care. Functional status serves as an indicator of independency and quality of life and functional limitations are detrimental to older patients since they trigger of the demand for health care, worsen quality of life, threaten independence, and increase the risk of nursing home placement and mortality [1, 2].

Older hospitalized patients are at an particularly high risk of functional decline during their hospital stay [3, 4]. Functional decline is described as a loss of independence in self-care activities or a deterioration in self-care skills. Usually, it is measured using a basic activities of daily living (ADL) scale. Such a scale encompasses activities like walking, transferring, dressing, eating, grooming, and using a toilet. Among various scales to assess ADLs, Barthel’s Index (BI) is the most widely used measure for the assessment of functional status [5]. The score of the BI ranges from zero to 100 with higher scores meaning lesser impairments. The minimal clinical important difference (MCID) of the BI as a measure of relevance of a difference was found to be about 20 points in a sample of stroke victims [6].

Overall, the risk factors of functional impairment in older adults are numerous and include—among others—old age, poor self-rated health, burden of diseases, life-style habits [7], and medication use [8]. Importantly, several of these factors are modifiable. Geriatric rehabilitation is a comprehensive and effective multi-component intervention with the intention to address most of these factors and to improve functional status in older individuals [9, 10]. The main components of geriatric rehabilitation are physical, occupational, and psychological therapy, controlling of pain, and improvement of nutritional status [10].

Based on numerous studies, the number of drug prescriptions increases with age as does the number of diseases [11–13]. Polypharmacy, defined as the prescription of five or more drugs in the same person is found in 44% of elderly men and 57% of elderly women in the US [14]. In a more recent study the overall prevalence of polypharmacy was nearly identical for women (32.1%; 95% CI 31.3–32.9) and men (32.2%; 95% CI 31.4–33.0) [15]. Due to the lack of evidence regarding safety and efficacy of many drugs in older adults, polypharmacy is often inappropriate and leads to adverse clinical outcomes such as functional impairment [16]. Especially frailty and functional impairment in older individuals are associated with a higher risk of unfavorable outcomes [17, 18].

So far, several studies showed an association between drug prescriptions and functional decline [19–24]. However, since all of these studies are observational or cross-sectional, a causal relationship between drug prescriptions and functional status is inconclusive. Up to now, there are no prospective and interventional randomized studies investigating the impact of the modification of drug prescriptions on functional status as an outcome in patients receiving in-hospital geriatric rehabilitation.

In addition, there is not much convincing evidence that the strategies in modifying drug prescription have an impact on clinically relevant endpoints. This is because interventions are complex and it is still unclear how to optimally organize and implement them to achieve a reduction in inappropriate polypharmacy [25].

The VALFORTA trial is a prospective randomized interventional study that investigated the impact of a FORTA (Fit-fOR-The-Aged)-guided prescription versus standard prescription care in older in-hospital patients [26]. In brief, the FORTA list is the only drug appropriateness list for older people that does not only label potentially inappropriate medications (PIM), but also those drugs that should be given and are potentially omitted medications (POM), it is, thus, the only positive-negative drug list for older people [22, 27–29]. The START/STOPP criteria provide guidance regarding PIM and POM as well, however, in contrast to the FORTA list they include both general action points and drug assessments, START/STOPP thus is no exclusive drug list like FORTA [29].

Here, we analyzed the data of a subsample of the VALFORTA trial. The impact of medication optimization according to the FORTA-list [27] on functional status was determined in geriatric patients hospitalized for rehabilitation as effects of FORTA seen in the entire cohort may be affected by rehabilitation, e.g., modified or even diluted by this structured and comprehensive intervention.

## Methods

The VALFORTA trial is a prospective randomized controlled trial that was conducted in two geriatric departments [26]. In short, inclusion criteria were age 65 years or over and three or more long-term medications (or age 60 years or over and 6 or more medications), hospitalization for five days or longer, multi-morbidity defined as three or more medical conditions at the same time, and written informed consent. Patients were randomized to standard geriatric care (control group) or additional management of medication according to the FORTA criteria (intervention group) [26, 28]. The primary end point of the VALFORTA trial was the FORTA score. The FORTA score is the sum of over- and under-treatment prescription errors according to the FORTA list. Consecutive patients were randomized to the intervention and control ward and the outcome assessment was blinded. In addition, an intention-to-treat analysis was used. The study protocol was approved by the Ethics committee of the Medical Faculty Mannheim, Heidelberg University, Germany, and the Ethics committee of the University of Witten-Herdecke, Germany. Further details about the study population are described elsewhere [26].

We collected personal data including age, gender, body-mass-index (BMI), number of diseases, number of medications on admission and discharge, number of applied occupational and physiotherapeutic units, and length of stay. The evaluation of the medication was conducted on admission and on discharge according to the FORTA criteria in both groups. Additionally, all subjects received a comprehensive geriatric assessment as a clinical routine on admission and the assessment of ADLs by means of BI, usual gait speed, and mobility by means of the Tinetti-test on discharge [26].

For this analysis, we selected patients of the Essen cohort who received geriatric rehabilitation which required a length of stay of at least 14 days. We choose the increase of the BI by at least 20 points on discharge as the main outcome variable. This cut-off value of the BI is considered as MICD (see above). In addition, the achievement of at least 70 BI points was analyzed since it characterizes the initially impaired geriatric patients as at most mildly affected on discharge [30]. Length of stay, age, gender, BI on admission, Charlson Comorbidity Index (CCI), number of applied treatment units and patient group were analyzed as independent variables.

### Statistical analysis

We used descriptive statistics and calculated percentages or median and interquartile range (IQR). The chi-square test was used for comparison of categorial variables. To assess the association between the gain of BI of at least 20 points and patient group we calculated Cramers V, the odds ratio and the power using G*Power 3.1.9.7 (Heinrich Heine University, Duesseldorf, Germany). Logistic regression analysis (a multivariable analysis) was used to assess the association between a gain of at least 20 BI-points and patient group as well as potential confounding variables including age, gender, CCI, number of treatment units applied, BI on admission, dementia and length of in-hospital stay. Statistical analyses were performed with IBM SPSS 28.0 (IBM Corporation, Armonk, NY, USA). A two-sided *p* < 0.05 was considered significant.

## Results

Out of a total of 246 patients study patients, we derived a subsample of 189 (73%) individuals with a length of stay of at least 14 days. Ninety-six (51%) patients—with *N*=27 (28%) males—belonged to the intervention group. The control group consisted of 93 (49%) patients with *N*=27 (29%) male patients (n.s.).

Table 1 shows the distribution by gender, the diseases, co-morbidities, the type of the referral to the geriatric unit, the level of care, and the number of patients with nursing home residency. As expected and typical for patient of a geriatric rehabilitation unit, the majority of the individuals had mobility disabilities, recent falls, and fall related fractures. Level of care was zero or mild (level 1) in most patients. Interestingly, in the intervention group the portion of patients which were referred from other hospitals was higher than in the control group. In contrast, the portion of community-dwelling patients was higher in the control group.

**Table 1:** Personal data of the subsample with geriatric rehabilitation

	All	Control	Intervention	*p* Value
	Number/% (*N*=189)	Number/% (*N*=93)	Number/% (*N*=96)	
Male	54/29	27/29	27/28	0.890
Main diseases				
Pulmonary disease	5/3	4/4	1/1	
Mild dementia	11/6	5/5	6/6	
Cardiovascular	18/10	9/10	9/10	
Mobility/falls	155/82	75/81	80/83	0.571
Type of referral				
Emergency	6/3	3/3	3/3	
GP	46/26	31/34	15/16	
Hospital	137/71	59/63	78/81	< 0.02
Level of care				
None	117/62	62/67	55/57	
1	61/32	25/27	36/38	
2	11/6	6/6	5/5	
3	0	0	0	0.294
Nursing home residency				
	12/6	6/6	6/6	0.955

*GP* General practitioner, *N* Total number of patients

Table 2 shows the results of the geriatric assessments. On admission, most patients were moderately compromised in the activities of daily living. Only a minority of patients suffered from dementia and dementia severity was usually mild.

**Table 2:** Results of the geriatric assessment, number of drugs and quality of medication according to the FORTA list

	Total	Control	Intervention	*p* Value
	*N*=189	*N*=93	*N*=96	
	Median/IQR	Median/IQR	Median/IQR	
Age [years]	84/79–87	82/78–85	86/81–89	0.001
BI on admission [0–100]	50/35–65	50/35–75	45/30–65	0.082
BI on discharge [0–100]	65/45–80	60/45–80	65/45–80	0.557
IADL [0–8]	4/2–7	4/2–6	5/3–7	0.054
MMSE [0–30]	27/24–28	27/24–28	27/24–29	0.448
Charlson comorbidity index [0–]	2/1–3	2/1–3	2/1–3	0.038
Number of drugs on admission [N]	7/5–9	8/6–9	7/5–9	0.249
Number of drugs on discharge [N]	8/7–10	9/7–10	8/6–10	0.549
FORTA score on admission	2/1–4	2/2–4	2/1–4	0.129
FORTA score on discharge	0/0–1	1/1–2	0/0–0	0.001

*BI* Barthel Index, *IADL* Instrumental Activities of Daily Living, MMSE Mini–Mental State Examination, *FORTA score* Sum of over- and under-treatment prescription errors according to the FORTA list

The number of drugs on admission was substantial. Furthermore, despite management in a geriatric unit, the number of drugs on discharge did not decrease as PIMs were replaced by POMs at comparable numbers. Yet, management according to the FORTA list led to an improvement in overall quality of medication as measured by the FORTA-score in both groups. Of note, the amount of improvement in medication quality was significantly greater in the intervention group.

On discharge, 79 (42%) patients reached a BI of at least 70 points, 46 (48%) patients of the intervention group and 33 (35%) patients of the control group (*p*< 0.09).

**Fig. 1:**
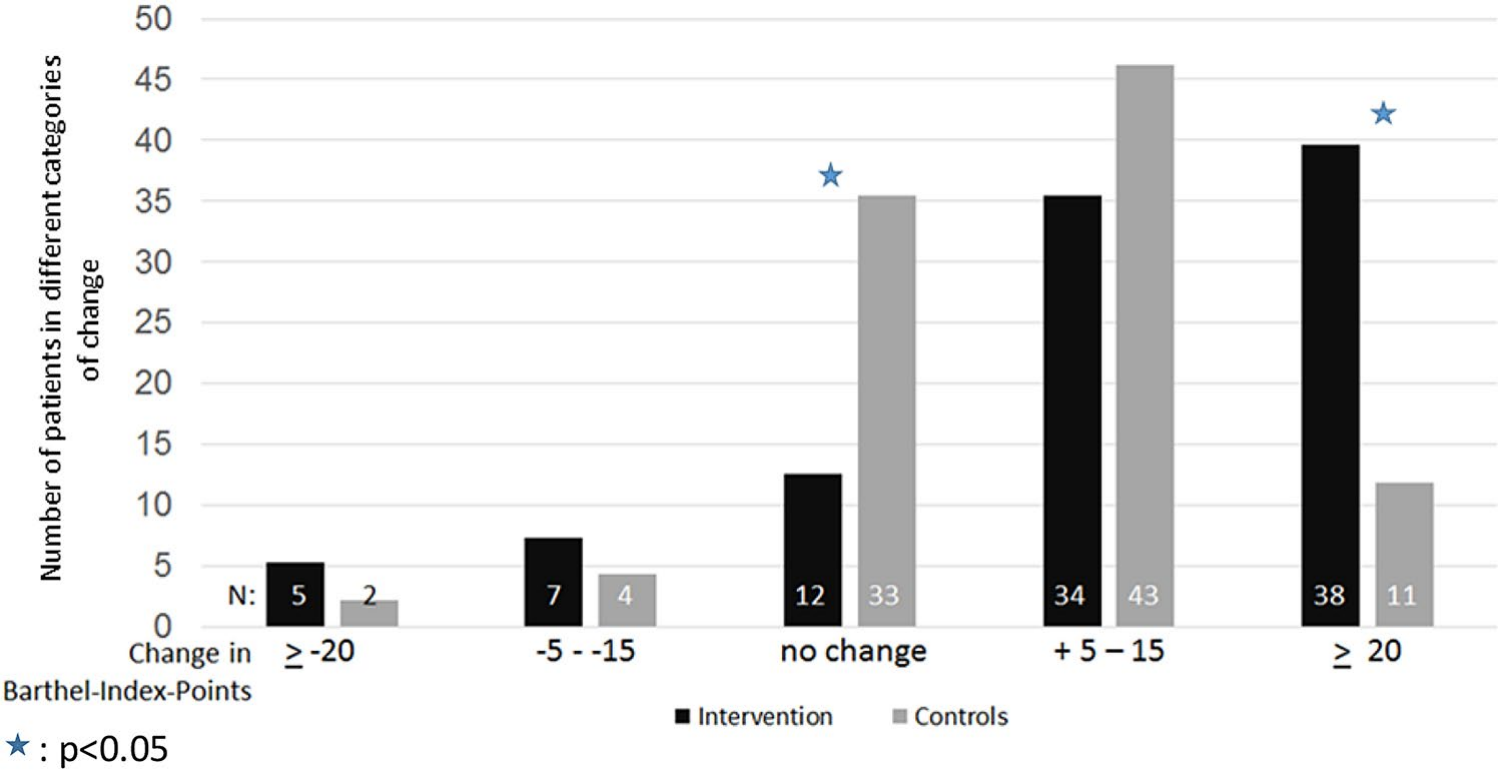
The comparison of degrees of changes in Barthel Index (BI) during the geriatric rehabilitation between the intervention and the control group. Blue shaded star: *p*<0.05

The comparison of different degrees of changes in BI during the geriatric rehabilitation is presented in Fig. 1.

An improvement in BI was found in in 72 (75%) patients of the intervention group an in 54 (58%) patients of the control group. However, the minimal important clinical difference (MICD) of an increase in the BI of at least 20 points was found in 49 (26%) of the whole sample, in 38 (40%) patients of in the intervention group and in 11 (12%) patients of the control group (*p*< 0.001).

The odds ratio for a gain of at least 20 BI points for patients of the intervention group was 2.89 (95% CI 1.465–5.703, *p*< 0.001) with a power of more than 80% and a value of Cramer-V of 0.317.

No Change of the BI or a deterioration of the BI was observed in 12 (13%) and 12 (13%) of the patients of the intervention group and in 33 (34%) and 6 (6%) control patients. The number of patients in the control group with no change in BI was significantly higher as compared to those in the intervention group (*p*<0.001).

Logistic regression analysis showed a significant and independent association between an increase of at least 20 BI-points and patient group (2.358, 95% CI 1.145-4.847, *p*< 0.02), BI on admission (0.957, 95% CI 0.939-0.976, *p*< 0.001 and the CCI (0.793, 95% CI 0.635-0.991, *p*< 0.041). No association was found for the number of treatment units, dementia, gender, length of stay and age.

In addition, falls occurred in 19/87 (22%) patients of the control group and in 11/75 (15%) patients of the intervention group (n.s.). However, there was a trend for rarer bipedal falls in the intervention group 4/75 (5%) compared to 9/87 (10%) bipedal falls in the control group (*p*< 0.094).

## Discussion

To the authors’ knowledge this is the first study that analyzed the impact of a prospective guided randomized intervention to optimize quality of drug prescription, under- and over-treatment on functional outcomes in older in-hospital rehabilitation patients.

The main result is the significant and independent association between the gain in functional status of at least 20 BI points—as a cut-off value assumed as the MICD—and the FORTA-guided improvement of the drug treatment. Though the VALFORTA trial was a prospective randomized intervention trial, this association found in a secondary analysis does not prove causality and needs to be corroborated in further clinical trials.

Activities of daily living is a main outcome parameter in geriatrics. The ability of attainment or conservation of independence is of outstanding importance, since it is also associated with quality of life.

Most interventions referred to cardiovascular diseases, osteoporosis and persistent pain. According to the FORTA concept the quality of prescriptions not only focused on over-treatment as it would be by the application of PIM lists such as the Beers list, but under-treatment is addressed as well [26]. Among other factors this may lead to better pain of blood pressure control for example, and overall improved physical endurance resulting in improved activities of daily living.

Of note, the number of subjects with dementia was low in our sample compared to a usual geriatric unit. Therefore, results cannot be generalized to all older subjects but only to patients with a comparable disease burden.

Observational studies consistently show the negative association between the anticholinergic drug burden and functional status. For instance, in a study of 418 older subjects, the anticholinergic risk burden was associated with lower functional improvement measured by means of the Functional Independence Measure (FIM) [31]. Since a large number of medications with a FORTA label D have anticholinergic activity, application of FORTA reduces the anticholinergic burden. This effect was more frequent in the intervention group considering the significantly lower FORTA score on discharge in this group. The results of this observational study are in line with our results.

In summary, observational studies support the evidence that improving the quality of prescribing and reducing over and under-prescriptions as well is associated with functional improvement [32, 33].

A meta-analysis that included 25 studies comprising 10980 patients in total analyzed the impact of strategies to reduce polypharmacy on clinically relevant endpoints like mortality or hospital admissions. As a main result, the authors could not find convincing evidence for a relevant clinical impact. However, in most the studies that were included in this meta-analysis functional or cognitive variables were not available as an outcome measure [25]. In brief, only 4 (A. G. Zermanysky et al., G. Pope et al., D. Frankenthal et al., and M. E. Williams et al.) [25] of the 25 trials included physical functioning, functional independence measure or the Barthel index as an outcome and in all four trials the intervention had no significant impact on these outcomes. Only one of these trials (G. Pope et al.) [25] was performed in patients in residential hospitals with continuous care wards and included a medication review by a multidisciplinary panel using the Beer’s Criteria, the Inappropriate Prescribing in the Elderly Tool and the British National Formulary to optimize the drug treatment in the intervention group. None of the trials dealt with the topic of this work, namely the impact of optimization of pharmacotherapy on geriatric rehabilitation.

The FORTA-guided intervention encompasses all three main interventional components: it addresses (a) over- and (b) under-treatment and supports the best choice of a prescription, thus avoiding, (c) mistreatment [26]. The overall impact of the FORTA-guided intervention on medication is measured by the FORTA score. The lower the FORTA-score the higher the quality of medication [26].

In addition, an amelioration of prescribing occurred in both groups of the subsample. This reflects the general impact of geriatric care. However, the impact was significantly greater in the intervention group. As already mentioned, several factors have an impact on functional outcomes in older people. One strength of this analysis is that most of these factors were available und could be included into the analysis.

Importantly, the patient group was independently and significantly associated with a favorable functional outcome. Moreover, the number of patients who were referred from a hospital to the geriatric ward was significantly higher in the intervention group and the patients in this group were significantly older as compared to the control group. Both attributes would be assumed to indicate worse outcomes, but the intervention was successful despite these disadvantages in the intervention group. This result underscores the need of a comprehensive management of prescriptions—as it is supported by FORTA. Furthermore, such a procedure seems to be more important than mere deprescribing of drugs. The additional integration of aspects of under- and over-prescribing seems to be a pivotal measure.

## Limitations

We conducted a secondary analysis of data of the VALFORTA study and only a subsample of patients with geriatric rehabilitation was included. The primary endpoint of the VALFORTA study was the impact of FORTA on the quality of medication but not on functional variables [26]. Therefore, the results of this secondary analysis must be interpreted with caution, and results of this analysis do not prove causality.

Furthermore, the patients of this analysis constitute a subgroup with a supposed potential for functional improvement by comprehensive geriatric rehabilitation which in turn may affect the intervention by FORTA. In addition, team-based treatment and the daily application of physiotherapy and occupational therapy belong to the basic concept of a geriatric unit. Therefore, the applicability of the findings to older patients treated in a usual medical ward is limited. However, since both groups did not differ in the therapeutic setting as a whole, a FORTA-guided intervention might also have a favorable impact on functional status in older people treated in other hospital units or nursing homes [34]. However, the results can constitute a basis for the formation of hypotheses that integrate functional parameters as outcome variables and emphasize quality of medication. Further prospective interventional studies are warranted to prove this assumption.

## Data Availability

The data used in this study can only be provided to other researchers upon submission of a written request and evaluation by our study group.
